# Gene-Interaction-Sensitive enrichment analysis in congenital heart disease

**DOI:** 10.1186/s13040-022-00287-w

**Published:** 2022-02-12

**Authors:** Alexa A. Woodward, Deanne M. Taylor, Elizabeth Goldmuntz, Laura E. Mitchell, A.J. Agopian, Jason H. Moore, Ryan J. Urbanowicz

**Affiliations:** 1 Department of Biostatistics, Epidemiology and Informatics, University of Pennsylvania, Philadelphia, PA USA; 2 Children’s Hospital of Philadelphia, Philadelphia, PA USA; 3 Human Genetics Center, Department of Epidemiology, Human Genetics and Environmental Sciences, UTHealth School of Public Health, Houston, TX USA; 4 Department of Computational Biomedicine, Cedars-Sinai Medical Center, Los Angeles, CA USA

**Keywords:** Gene set enrichment analysis, GWAS, Epistasis, Congenital heart disease

## Abstract

**Background:**

Gene set enrichment analysis (GSEA) uses gene-level univariate associations to identify gene set-phenotype associations for hypothesis generation and interpretation. We propose that GSEA can be adapted to incorporate SNP and gene-level interactions. To this end, gene scores are derived by Relief-based feature importance algorithms that efficiently detect both univariate and interaction effects (MultiSURF) or exclusively interaction effects (MultiSURF*). We compare these interaction-sensitive GSEA approaches to traditional *χ*^2^ rankings in simulated genome-wide array data, and in a target and replication cohort of congenital heart disease patients with conotruncal defects (CTDs).

**Results:**

In the simulation study and for both CTD datasets, both Relief-based approaches to GSEA captured more relevant and significant gene ontology terms compared to the univariate GSEA. Key terms and themes of interest include cell adhesion, migration, and signaling. A leading edge analysis highlighted semaphorins and their receptors, the Slit-Robo pathway, and other genes with roles in the secondary heart field and outflow tract development.

**Conclusions:**

Our results indicate that interaction-sensitive approaches to enrichment analysis can improve upon traditional univariate GSEA. This approach replicated univariate findings and identified additional and more robust support for the role of the secondary heart field and cardiac neural crest cell migration in the development of CTDs.

**Supplementary Information:**

The online version contains supplementary material available at (10.1186/s13040-022-00287-w).

## Introduction

Gene set enrichment analysis (GSEA) has emerged as a useful approach to hypothesis generation. While not a deterministic strategy for identifying associations, GSEA is often applied to pursue interpretation of the functional significance of genetic data and to prioritize signals for downstream analysis [1]. GSEA was originally developed for use with gene expression data,[1] but many extensions to the method allow for use of SNP-level data, and in some cases, GWAS summary statistics [2]. Genes are assigned to gene sets using annotation databases such as the Kyoto Encyclopedia of Genes and Genomes [3] and the Gene Ontology (GO) resource [4, 5]. Enrichment analyses are typically conducted using either self-contained or competitive hypothesis testing [6]. The latter of the two tests the magnitude of phenotype association of genes in a gene set in contrast to the rest of the genes in the genome. This study focuses on competitive testing.

In the context of the ‘common-disease common variant hypothesis’ and the small effect sizes for most individual variants [7], GSEA is powered to detect genetic risk factors via consideration of the collective effect of multiple variants within the same gene set. GSEA may also be particularly useful in the presence of genetic heterogeneity where more than one genetic mechanism results in the same, or similar, phenotype/disease [8]. However, standard GSEA approaches preclude the consideration of complex gene-gene interactions (i.e. epistasis). Modern perspectives regarding complex human disease often note the importance of complex genetic architectures including both genetic heterogeneity and epistasis [9–12]. Others have shown that it is important to consider the potential impact of these complicating phenomena and to develop and adopt methodologies capable of taking them into account [13]. Thus, substantial information could be gained by capturing interactions in addition to univariate effects prior to GSEA.

Despite their potential importance, epistatic interactions can be notoriously difficult and computationally expensive to search for and detect [14]. Some successful methods rely on exhaustive examination of candidate variable pairs or sets, (e.g. Multifactor Dimensionality Reduction [15]). This can be computationally prohibitive as (1) the size of the feature space, i.e. the number of variants, genes, or other variables becomes very large, or (2) higher order interactions are sought, e.g. 3-way, 4-way, etc.

Relief-based algorithms (RBAs) are a family of filter-based feature importance estimation methods that are unique in their ability to detect epistatic interactions without an exhaustive search of every *p*^2^ (two-way) or higher-order interaction [16]. RBAs achieve this through a feature scoring heuristic operating on pairs of samples that are maximally similar to one another. These algorithms scale linearly with the number of features but quadratically with the number of samples [16]. This has made them popular in genomic analyses which are often characterized by large feature spaces but relatively small sample sizes [17, 18]. The first RBA was proposed by Kira and Rendell in 1992 [19] and has since spawned many algorithmic variants as reviewed in [16]. Recent research using simulated data introduced and identified MultiSURF to be the most effective and flexible RBA (to date) for detecting simple univariate effects as well as both pure 2 or 3-way epistatic interactions [20]. That same study demonstrated that MultiSURF* [21] was somewhat more effective at detecting epistatic interactions, however this was at the expense of being able to detect univariate associations. This makes MultiSURF* an effective approach to exclusively search for features that contribute to interaction effects.

In the present study we compared traditional univariate metrics and statistics with RBA feature importance scores for gene ranking prior to GSEA. We hypothesized that conducting GSEA with a gene ranking that takes epistatic interactions into account will improve the identification of relevant biological themes/pathways and lead to novel hypotheses. We first test this hypothesis in a smaller simulated dataset that included multiple pairwise interactions. We demonstrate the efficacy of using RBA feature scores for ranking in real-world data by comparing (1) univariate analysis ranking, (2) MultiSURF ranking, and (3) MultiSURF* ranking, in concert with GSEA using genome-wide genotype data from two cohorts with congenital heart disease (CHD) as the target disease phenotype. Future work will also seek to demonstrate generalizability of the approach to other complex diseases and data types.

CHD is a genetically heterogeneous disease and the most common birth defect in infants, with a prevalence of approximately 8 per 1000 live births and is among the leading causes of infant mortality [22, 23]. In non-syndromic CHD, a variety of single nucleotide and copy number variants and environmental factors are associated with disease risk [24]. However, in the majority of cases, the exact cause remains unknown [25]. Conotruncal defects (CTDs) are a highly heritable and common subgroup of CHD that affect the cardiac outflow tract (OFT) and include tetrology of Fallot, d-transposition of the great arteries, and other malformations [26]. Development of the OFT during cardiogenesis is well characterized [27–29]; involving complex, time-dependent, and interacting processes. A better understanding of the genes and pathways influencing progenitor cell behavior would improve our understanding of CTD etiology. A few studies have directly interrogated epistasis in the folate metabolism pathway as a risk factor for CTD [30], however to our knowledge, no CTD studies have performed a more comprehensive analysis of interaction across many genetic variants, genes, or pathways.

## Methods

**Fig. 1:**
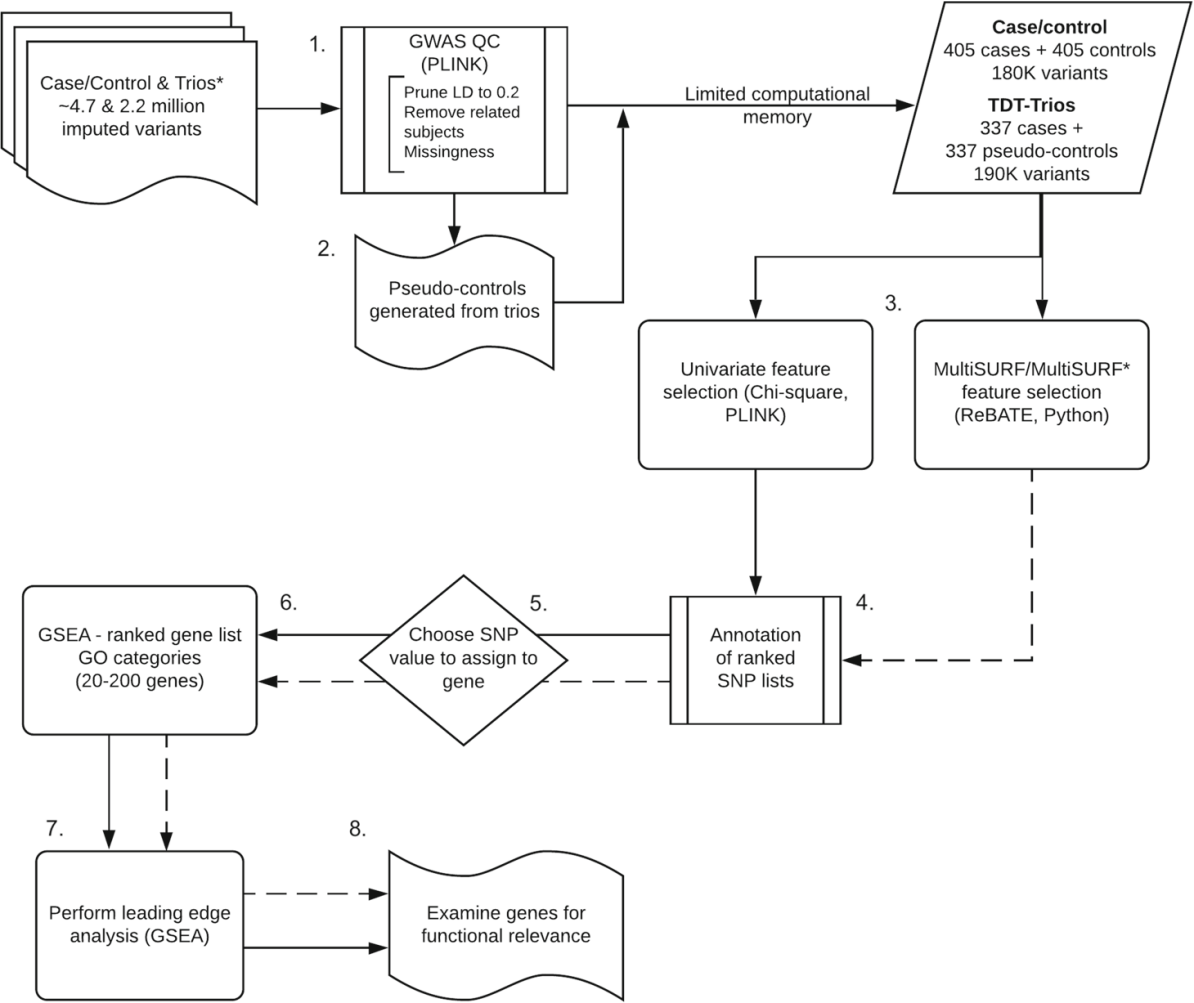
Flowchart of the univariate and Relief-based methods applied to the case-control and trio data

In this section we describe how the simulated data were created and how the CHD data were utilized and pre-processed. We also provide a detailed overview of the analysis pipeline in which enrichment analysis is preceded by either traditional univariate analysis or interaction-sensitive RBA analysis for both the simulation study and the CHD data. The steps in this analysis pipeline are outlined in Fig. 1.

### Simulated data

Simulated genome-wide array data were created using GAMETES v2.2, [31] with individual genotypes coded 0/1/2. To generate SNPs with no effect, we first randomized the genotypes for 1000 SNPs using the CHD array data from 405 cases and 405 controls. Next, we generated 8-feature additive 2-way epistasis, corresponding to four pairs of interacting SNPs that contribute additively to the outcome, i.e., each pair contributes one-fourth of the effect. All SNPs in the epistatic pairs had minor allele frequency (MAF) of 0.2 and heritability of 0.4. SNPs with no effect were randomly assigned a subset of rsIDs from the CHD data. The pairs of epistatic SNPs were assigned rsIDs from 8 genes in the GO category *negative regulation of mRNA splicing*; PTBP1, U2AF2, SRSF9, SFSWAP, PCBP4, NPM1, C1QBP, and SAP18. The SNPs were ranked using the chi-square statistic obtained from the comparison of the genotype distributions in cases and controls, MultiSURF, and MultiSURF* and assigned to genes (steps outlined below).

### Congenital heart disease data

This project utilized de-identified array and imputed genotype data from two independent cohorts recruited under identical protocols at The Cardiac Center at the Children’s Hospital of Philadelphia: (1) a discovery cohort of 406 CTD cases and 2,976 controls (Cohort 1)and (2) a replication cohort of 317 CTD case-parent trios (Cohort 2). All samples were array genotyped using Illumnia arrays [32]. Full details on recruitment, including inclusion and exclusion criteria and patient characteristics have been previously described [32].

#### Data quality control

Standard quality control procedures had been implemented in PLINK v1.06 for both study cohorts, as previously described [32]. Briefly, for both datasets, SNPs with a minor allele frequency < 1*%* or genotyping rate < 90*%*, and cases/trios with a Mendelian error rate > 1*%* or pairwise identity-by-descent > 0.6 were excluded. Genotypes were imputed using Impute 2 v2.3.0, and poorly imputed or rare (MAF < 5*%*) variants were removed post-imputation.

#### Cohort 1

To reduce both class bias and the computational requirements to run the RBA algorithms (which scale quadratically with the number of samples) [16], we used an equal number of cases and randomly selected controls. One case was removed after a pairwise identity-by-decent analysis suggested a second-degree familial relationship, resulting in 405 cases and 405 controls. Further, as high LD can result in increased bias [33, 34], we used a strict LD pruning threshold of *r*^2^ = 0.2 (window size of 50 and step of 5) to reduce the 4.7M genotyped and imputed SNPs (Fig. 1, step 1.). A total of 184,526 SNPs were carried forward for feature ranking.

#### Cohort 2

**Fig. 2:**
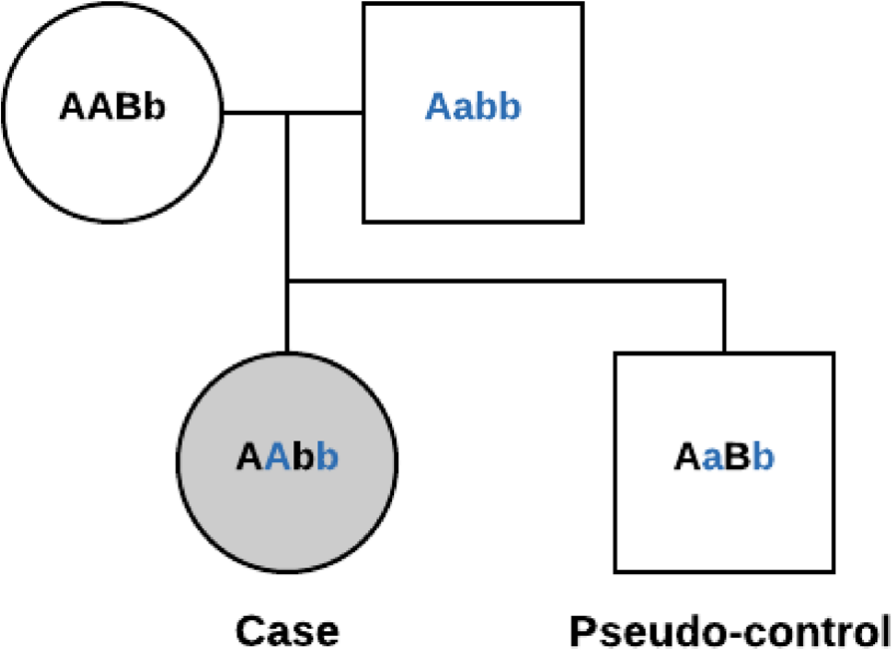
Example case and pseudo-control generated from a case-parent trio

Our replication dataset consisted of 337 case-parent trios. With 2.2M SNPs in the original dataset, LD pruning was performed in the same manner as above, resulting in 193,354 SNPs (Fig. 1, step 1). We used the “–tucc” command, implemented in PLINK v1.09, to create pseudo-controls based on the parental alleles that were not transmitted to the affected case (Fig. 2) [35]. The RBA feature ranking algorithms used in this analysis require the outcome to be in a binary (case-control) format.

#### Data formatting

PLINK binary files for both datasets were re-coded to produce a single text file with additive SNP genotypes coded in the standard 0/1/2 format for the number of variant alleles. Columns were filtered to keep only the rsIDs, and binary phenotype value.

### Feature ranking

We compared univariate and Relief-based approaches for SNP-level feature ranking in preparation for GSEA. The following steps were performed for both the discovery and replication cohorts (Fig. 1, steps 3-8).

#### RBA ranking

After creating 10-fold cross-validation datasets, we used the skrebate package in scikit-learn (Python v3.7.1) to implement two RBAs, MultiSURF and MultiSURF*, to generate feature importance scores for each SNP (Fig. 1, step 3). Both algorithms score features by comparing instance pairs with feature values that are maximally similar or dissimilar. Features with different values between a given instance pair have their scores positively or negatively updated based on whether the outcomes are respectively different or the same. Collective feature scores derived over the entire set of samples are normalized to fall in the range [-1,1], with higher scores indicating greater importance in predicting outcome. This scoring approach allows RBAs to indirectly detect interactions between features without the need for an exhaustive search. Neither algorithm requires hyperparameter tuning or optimization. The SNP feature scores from the 10 cross-validation sets were averaged to produce a single score for each SNP. Of note, RBAs are typically used for feature selection prior to modeling [20], however, this project used the feature weights for ranking rather than selection. Additionally, RBAs do not perform a large number of independent tests in the same manner as the *χ*^2^ approach, and thus are not subject to the burden of multiple testing corrections.

#### Comparative approach: univariate ranking

We performed a standard case-control association analysis in PLINK, generating chi-square statistics for each SNP to be used for ranking (Fig. 1, step 3). We compared the results of the univariate GSEA with the interaction-sensitive RBA approach.

### SNP-to-gene annotation and score assignment

The R (v4.0.1) package snpGeneSets [36] was used to annotate SNPs to genes using GRCh37/hg19 assembly. SNPs were assigned to genes using 10kb windows upstream and downstream. Each gene has n SNPs, and a summary score or statistic *S*_*i*_ where *i*=1...*n*, must be chosen for each gene. Similar to the approach by [37], for both the RBA and univariate approaches, the summary score or statistic for a given gene is the maximum *S*_*i*_.

### Gene set enrichment analysis

The ranked list from each approach was loaded into GSEA Pre-ranked (MIT/Broad Institute) software v4.0.2 [1, 38]. The pre-ranked analysis used the ranked list to calculate enrichment scores using a running sum statistic [1]. Permutations to account for multiple testing were done by gene set. All available gene sets with 20 - 200 genes from the most recent MSigDB release [1, 39], v7.1 corresponding to Gene Ontology (GO) [4] terms were used for this competitive enrichment analysis. To evaluate the overlap between leading edge subsets of the top GO terms, we used the Leading Edge Analysis tool within GSEA. Leading edge genes are a subset of the genes in a particular category that appear prior to the peak score and contribute the most to the enrichment score.

## Results

**Fig. 3:**
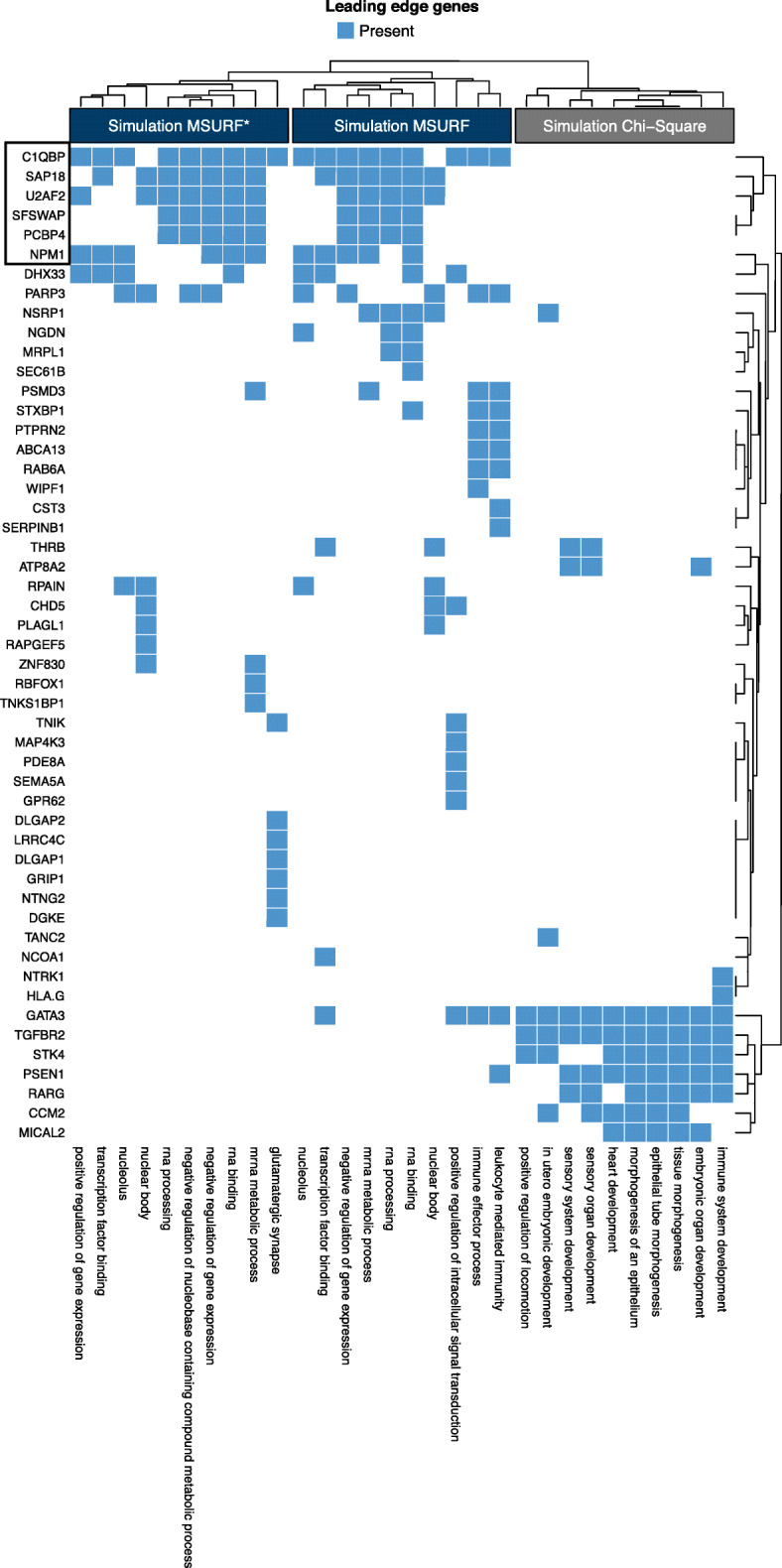
Heatmap of leading edge genes from the top 10 GO terms for each analysis of the simulated data. Hierarchical clustering was applied to both the rows and columns

Primary results from the simulation analysis include the top 10 GO terms and leading edge gene, illustrated in Fig. 3. For the CHD data, we include the top 15 GO terms identified by the pre-ranked GSEA for each of the three analysis strategies, i.e., univariate, MultiSURF and MultiSURF*, applied to both cohorts (Fig. 1, step 6). A full list of all false-discovery rate significant (FDR adj. P < 0.05) GO terms can be found in the supplementary material (Supplementary Table 1). Results from leading edge analyses of the top 10 GO terms for each of the six analyses further contextualize these significant GO terms (Fig. 6). We highlight common themes within and across the two datasets, in addition to similarities and key differences between the univariate and RBA analyses. We specifically discuss the evidence for interaction and main effects given by the MultiSURF and MultiSURF* results.

### Simulation results

Among the simulated data, only the interaction-sensitive GSEA approaches using MultiSURF and MultiSURF* were able to identify related pathways and the genes assigned to the pairwise interactions. Figure 3 shows the results of a leading edge analysis, highlighting the genes enriched in the top 10 GO terms from each analysis. The first six genes in Fig. 3 (top left) were part of the simulated interactions. Of the four epistatic pairs from the simulated data, MultiSURF and MultiSURF* captured two pairs and one gene each from the other two pairs. Further, the top 10 GO categories from the MultiSURF approach and the top nine from the MultiSURF* met the FDR significance threshold (adj. p < 0.05). Among the top categories were *rna binding*, *rna processing*, and *negative regulation of gene expression*, which shared genes in common with the pathway used for the simulation, GO category *negative regulation of mRNA splicing*. None of the *χ*^2^ results met the FDR threshold.

### Correlation analysis

**Fig. 4:**
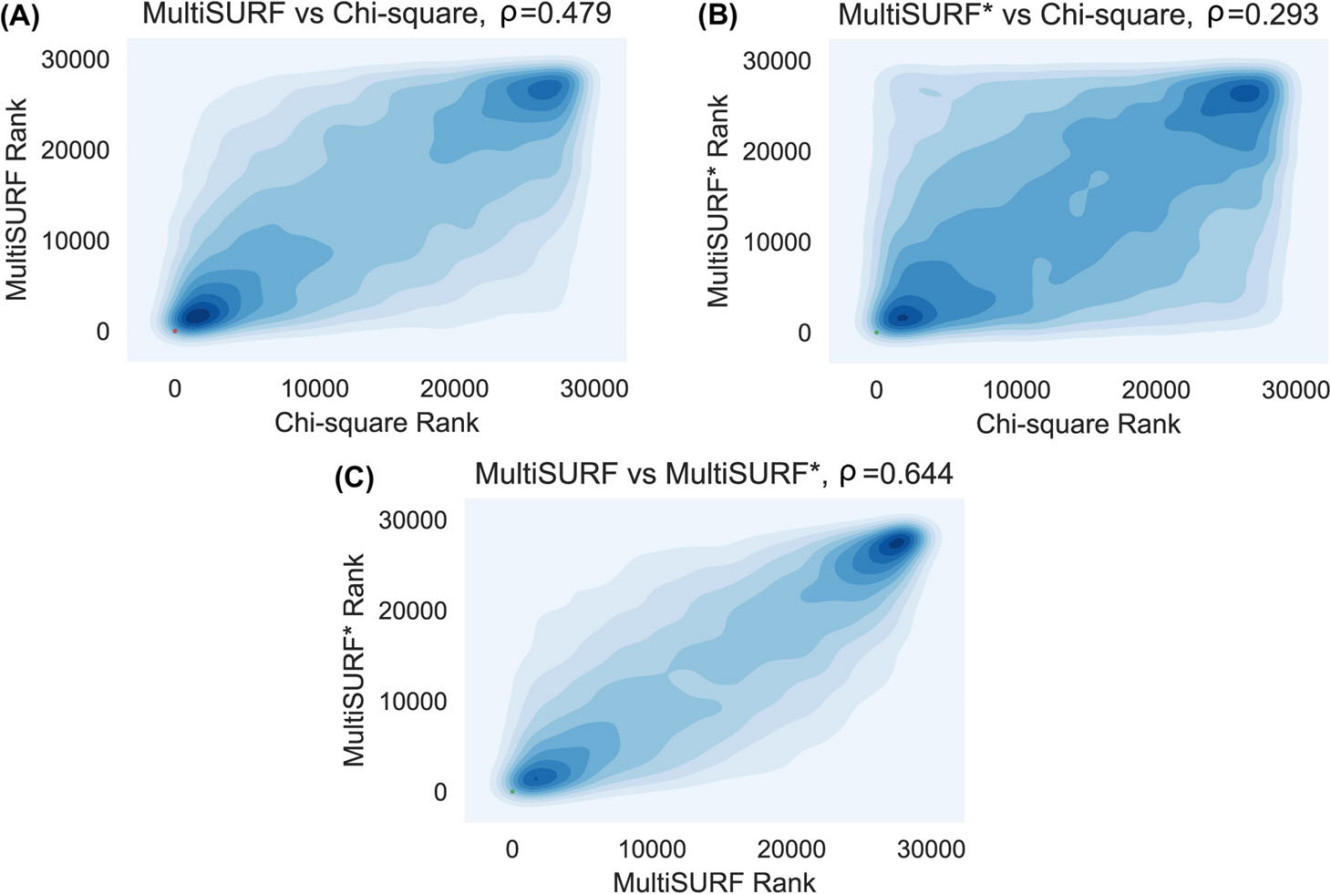
Density plots depicting the correlation between the gene ranks across the three analyses in Cohort 1. Spearman’s rank-order correlation coefficient (*ρ*) is given for each comparison

For the CHD data, we evaluated the overall correlation between the gene ranks for each analysis in both cohorts, all of which were highly significant (p < 0.001, Table 1). As expected, the univariate and MultiSURF results (Fig. 4a) were more highly correlated than the univariate and MultiSURF* results (Fig. 4b), given that MultiSURF captures *both* main effects and interactions and MultiSURF* captures interactions only. We also compared gene ranks from MultiSURF and MultiSURF*, and found a high level of agreement in both cohorts (Fig. 4c). This is suggestive of a greater contribution from interaction effects over univariate effects given that the highest gene rank correlation was found between the algorithm that can only detect interactions and the one that detects both univariate and interaction effects.

**Table 1:** Spearman’s rank-order correlation coefficients (*ρ*) for gene ranks between analyses

Data	*χ*^2^ & MultiSURF	*χ*^2^ & MultiSURF*	MultiSURF & MultiSURF*
Cohort 1	0.479	0.293	0.644
Cohort 2	0.543	0.325	0.737

### Univariate analyses

**Fig. 5:**
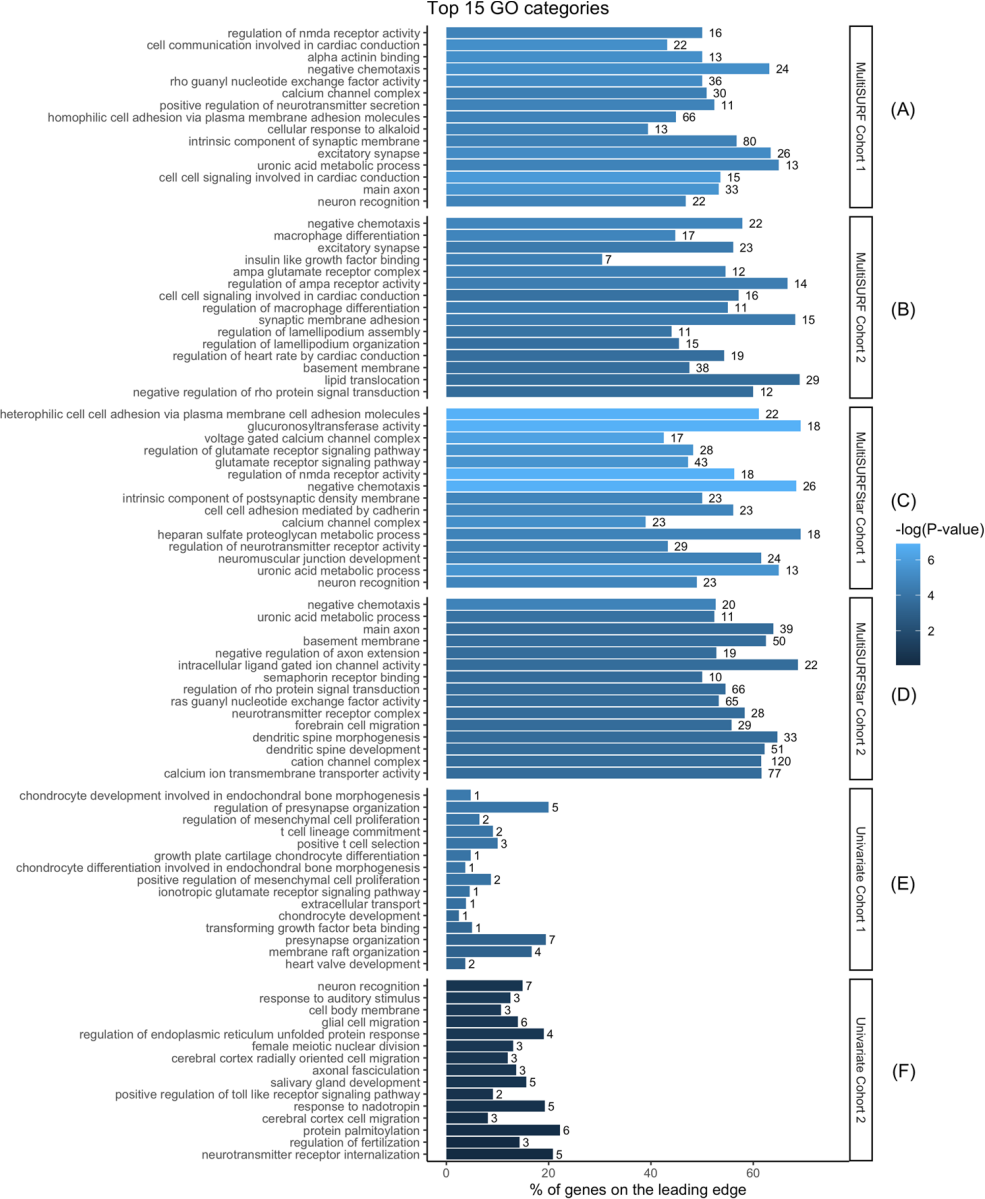
For each analysis, the bar plot illustrates the percentage of genes in each category that appeared on the leading edge. Numbers to the right of each bar are the number of genes on the leading edge for that category

Figure 5e details the top gene sets from the univariate analysis, where the top 15 GO terms from Cohort 1 (C1) met the FDR significance threshold (adj. p < 0.05). Two of these GO terms, *regulation of mesenchymal cell proliferation* (adj.p = 0.018) and *positive regulation of mesenchymal cell proliferation* (adj. p = 0.02) may reflect the role of the epithelial to mesenchymal transition[40] during OFT development. Two GO terms related to cardiac valve development, *heart valve development* (adj. p = 0.047) and *atrioventricular valve development* (adj. p = 0.051) also were among the top univariate results. Other pathways of interest outside of the FDR significance but within the top 40 GO terms include multiple GO terms related to neuronal development and migration and neural/vascular branching are suggestive of main effects, and also overlap with key themes in the RBA results. These pathways include *central nervous system projection neuron axonogenesis* and *branching involved in blood vessel morphogenesis*. To evaluate the robustness of our initial results, we repeated the univariate GSEA using cases and pseudo-controls generated from the case-parent trios (Cohort 2, C2). While none of the GO terms from the trio univariate GSEA met the FDR significance threshold, nominally significant (p < 0.05) pathways suggest some overlap with Cohort 1 results, including *neuron recognition*, *cerebral cortex cell migration* and *axonal fasciculation* and GO terms related to.

### RBA results

All of the top 15 GO terms from the RBA analyses met the FDR threshold (Fig. 5a-d, Supplementary Table 1). Multiple GO terms overlapped between the RBA analyses; for example, among the Cohort 1 results, 4 terms exactly overlapped with the MultiSURF (M) and MultiSURF* (M*) analyses: *Negative chemotaxis* (adj. p = 0.005, M-C1, 0.001 (M*-C1)), *neuron recognition* (adj. p = 0.009 (M-C1), 0.008 (M*-C1)), *regulation of NDMA receptor activity* (adj. p = 0.009 (M-C1)), 0.001 (M*-C1)), *uronic acid metabolic process* (adj. p = 0.009, M-C1), 0.004 (M*-C1)) (Fig. 5). As discussed further below, themes that appear in both M and M* results most likely represent interactions that may play a role in contributing to CTDs. Further, replication of themes across datasets offers supporting evidence that the significant GO terms reflect valid findings. Three GO terms replicated between the MultiSURF case-control and trio analyses: *excitatory synapse*, *cell-cell signaling involved in cardiac conduction*, and *negative chemotaxis*. One GO category and multiple GO themes replicated in the two M* analyses, including *negative chemotaxis* and calcium/cation channel complex and activity. Chemotaxis pathways have been previously captured in copy-number variant studies of CHD, [41, 42] notably playing a role in the development of the secondary heart field (SHF) [43].

GO terms related to synapse and neuron development appeared among the top 15 GO terms across all of the RBA GSEA, including *intrinsic component of synaptic membrane* (adj. p = 0.01 (M-C1)), *excitatory synapse* (adj. p = 0.006 (M-C1), 0.014 (M-C2)), *main axon* (adj. p = 0.028 (M*-C2), 0.004 (M-C1)), and *neuron recognition* (adj. p = 0.009 (M-C1), 0.008 (M*-C1)). This may reflect a number of shared factors that control the patterning of both the nervous and vascular systems, namely the semaphorin, netrin, and slit families and their receptors [44, 45]. These genes mediate axonal guidance [44] and the migration of neural crest cells via chemoattractive or repellent activity [46] [45]. Additionally, mouse models of plexin mutants (a semaphorin receptor) demonstrate OFT defects [47], as do avian *SEMA3C* mutants [48]. Many of these genes are also represented in the *negative chemotaxis* category, which was highly significant across all four RBA GSEAs.

Other significant gene ontology themes common between the four RBA analyses include cell-cell adhesion and cell signaling. Alpha actinin binding (adj. p = 0.005, M-C1) is implicated in cardiomyopathy,[49] and related genes have been also been reported in CHD [50]. Genes involved in Rho protein signaling, captured in both RBA analyses in Cohort 2, have been shown to impact cardiac looping and chamber maturation in mammalian systems[51], in addition to cell adhesion between cardiomyocytes and cardiac neural crest cell migration during embryogenesis [52, 53]. Other genes from cell adhesion pathways influence early cardiac development, including cadherins, mediated by the Wnt/ *β*-catenin signaling during embryonic gastrulation [54], integrins, [55] and nexins [42, 56]. GO terms related to the synthesis of heparan sulfate were significant in multiple analyses: *heparan sulfate proteoglycan metabolic process*, (adj. p = 0.009, M-C1), and *uronic acid metabolic process* (adj. p = 0.009 (M-C1), 0.001 (M*-C1), 0.021 (M*-C2)). Heparan sulfate plays a role in cell polarity and migration, [57] and studies in mice have shown that heparan sulfate biosynthesis can affect OFT development via downregulation of *EXT1* influencing the OFT progenitors (SHF and CNCCs) and disrupted Wnt/ *β*-catenin and FGF signaling [58–60]. Alongside *FGF8*, Notch signaling is known for its role in the SHF [61] and as a regulator of neurogenesis, [62] a similar theme among the top pathways and genes discovered in this analysis [44]. *NOTCH1*, *NOTCH2*, *FGF8*, *TGFB2* and *TBX1* are represented among the FDR significant GO terms in the case-control data including *outflow tract septum morphogenesis* (adj. p = 0.019 (M-C1), 0.044 (M*-C1)), *pulmonary valve development* (adj. p = 0.032, M-C1), *positive regulation of heart growth* (adj. p = 0.042 (M*-C2) *aortic valve development* (adj. p = 0.033, M-C1), and *notch binding* (adj. p = 0.038, M*-C1) [63].

### Leading edge analysis

Using a leading edge analysis, we were able to better understand which genes from these top GO terms may be most relevant in cardiac development, and which genes from these most significant GO terms replicate across analyses and in both datasets. The two univariate analyses shared one leading edge gene in common from the top 10 GO terms, *SHH*, a gene known for its role in guiding atrial septation in the SHF. [64, 65]. Other studies on CHD have also captured the hedgehog pathway [66] and demonstrated its necessity in cardiac neural crest cells during OFT development [67].

**Fig. 6:**
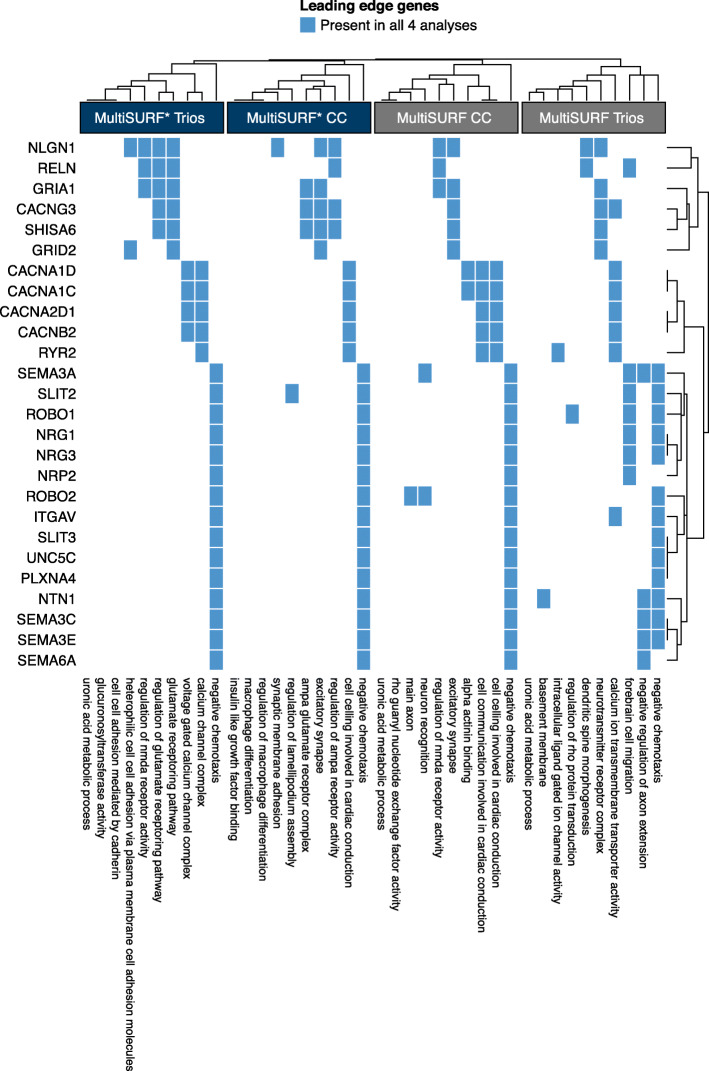
Heatmap of leading edge genes captured in all four of the Relief-based methods applied to the case-control and trio data. Hierarchical clustering was applied to both the rows and columns

Leading edge genes from the top 10 MultiSURF and MultiSURF* gene sets for both Cohort 1 and Cohort 2 are shown in Fig. 6. The figure is restricted to leading edge genes replicated across all four analyses. (A full list of leading edge genes from the top 10 gene sets from the RBA analyses can be found in Supplementary Table 2). Twenty-six genes (Fig. 6, Supplementary Table 3) replicated across all four RBA analyses, many of which have been implicated in cardiac development and CHD. They include *SEMA3C*, *SEMA3E*, *SEMA6A*; semaphorin receptors *NRP2* and *PLXN4A*; *SLIT2*, *SLIT3*, *ROBO1* and *ROBO2* from the Slit-Robo pathway, and *Integrin-* *αV*. Overall, the RBA analyses showed higher leading edge signals (Fig. 5. Across the top 15 terms, there were an average of 55% of the genes on the leading edge in the RBA analyses and 11% in the univariate analyses. This likely reflects the overall lack of significant SNPs (and thus genes) from the univariate association analysis compared to the RBA analyses.

## Discussion and conclusions

We have described a novel, interaction-sensitive approach to GSEA using Relief-based algorithms (RBAs). RBAs offer a computationally efficient approach to ranking features involved with underlying epistatic interactions. We evaluated our proposed methodology utilizing simulated data and genome-wide SNP data from patients with CTDs, a genetically heterogeneous group of congenital heart defects. In both the simulated and real-world data, we demonstrated the utility of this new methodological approach in capturing significantly enriched and biologically relevant GO terms and genes in contrast with traditional univariate GSEA. Further, we generated cases and pseudo-controls from trios for a replication dataset and saw similar themes of cell signaling, cell adhesion, and axon/neuron development and extension replicated across analyses. These GO terms highlight the common factors that guide neural and vascular patterning. Leading edge analyses further confirmed the enrichment of genes related to the SHF and CNCC migration in OFT development. The role of these cells in causing OFT developmental defects has been studied in mouse and avian models, but less extensively in humans. Non-syndromic cases of CTD present an opportunity to further investigate the genetic drivers of impaired development and migration of these progenitors [68]. Our approach to GSEA included a consideration of interaction at the SNP level, accounting for genetic interaction within or between genes. We speculate that the significant terms related to CNCC migration and progenitor behavior in the SHF captured by the Relief-based analyses reflect interactions between key signaling genes, such as those documented between interactions between *NOTCH* and *VEGFR*, [62] and *TBX1* and *FGF8* [69] that play a role in the development of CTDs.

The limitations of this approach are consistent with those from traditional univariate analysis based GSEA. First, we utilized LD pruning to produce a computationally tractable number of SNPs, but other methods such as using exon/coding regions could also be used. However, using exon/coding regions also has limitations, as it could leave out key intergenic regulatory regions. Secondly, SNP-based GSEA has the potential to be affected by gene size bias, whereby larger genes with more SNPs are more likely to have significant SNPs by chance alone. Additionally, our interaction-sensitive RBA approach to ranking SNPs does not specify which SNPs are involved in an interaction. Instead this needs to be inferred by examining differences in findings between MultiSURF* (which only detects interaction effects) and findings from univariate or MultiSURF based analyses. Determination of which pairs or sets of SNPs are interacting would require additional downstream analysis with statistical or machine learning modeling. There are known limitations in the ability of RBAs to detect pure interactions (i.e. no information can be gained by looking at informative features on their own) in very large feature spaces (e.g. over 100K features) [20]. However RBA wrapper algorithms such as TuRF [70] have been developed to help address this issue. Future efforts will examine the use of these RBA wrapper methods to determine the scalabilty of this interaction-sensitive GSEA approach in whole exome sequencing and whole genome sequencing data where feature spaces can greatly exceed 100K features.

These results suggest that interaction-sensitive GSEA offers the potential for generating new hypotheses and future research directions. Specifically, this approach can be used to prioritize genes or pathways for rare variant analyses or functional validation experiments. Future work will aim to replicate these findings in other independent CHD cohorts and apply this approach to other complex diseases.

## Supplementary Information

**Additional file 1** Density plots depicting the correlation between the gene ranks across the three analyses in Cohort 2. Spearman’s rank-order correlation coefficient (*ρ*) is given for each comparison.

**Additional file 2** Significant GO categories (FDR adj. p < 0.05) for each analysis, MultiSURF, MultiSURF*, and Univariate in Cohort 1 and Cohort 2. Results from each analysis is on an individual excel tab.

**Additional file 3** List of leading-edge genes that appeared on the leading edge of the top 10 gene sets in one or more of the RBA GSEAs. The values in each column are the number of times that gene appeared on the leading edge in that analysis.

**Additional file 4** Leading edge genes that replicated across all four RBA analyses.

## Data Availability

The data used for the analyses were derived from two studies. Cohort 1 has been registered through dbGaP and the data on the controls been uploaded to: https://www.ncbi.nlm.nih.gov/projects/gap/cgi-bin/study.cgi?study_idp̄hs000490.v1.p1 dbGaP Study Accession: phs000490.v1.p1 Data on cases from Cohort 1 and Cohort 2 (trios) are being prepared for submission to the related dbGaP project (which currently contains only summary statistics): https://www.ncbi.nlm.nih.gov/projects/gap/cgi-bin/study.cgi?study_idp̄hs000881.v1.p1 dbGaP Study Accession: phs000881.v1.p1 The GAMETES v2.2 software can be dowloaded at: https://github.com/UrbsLab/GAMETES/tree/v2.2.

## Abbreviations

CHD: Congenital heart disease
CTD: Conotruncal defects
C1, C2: Cohort 1, Cohort 2
EMT: Epithelial to mesenchymal transition
GSEA: Gene set enrichment analysis
GWAS: Genome-wide association study
GO: Gene ontology
M: MultiSURF
M*: MultiSURFStar
OFT: Outflow tract
RBA: Relief-based algorithm
SNP: Single nucleotide polymorphism
