# Supplementary figures and images for: Gene-Interaction-Sensitive enrichment analysis in congenital heart disease
Source: BioData Min. 2022 Feb 12;15:4. doi: 10.1186/s13040-022-00287-w (PMC8841104; doi:10.1186/s13040-022-00287-w)

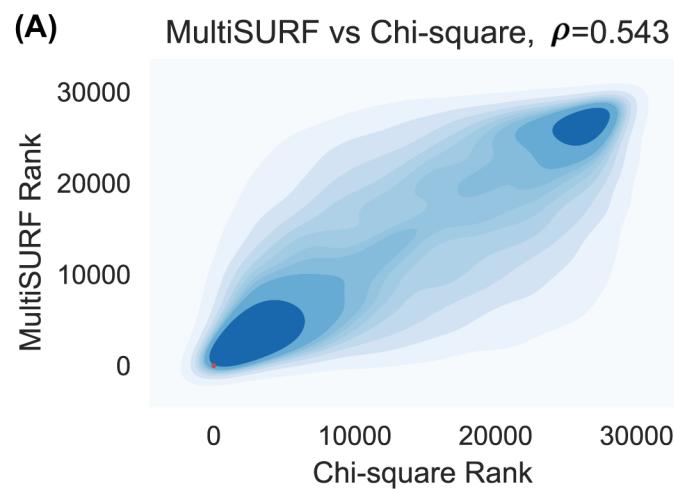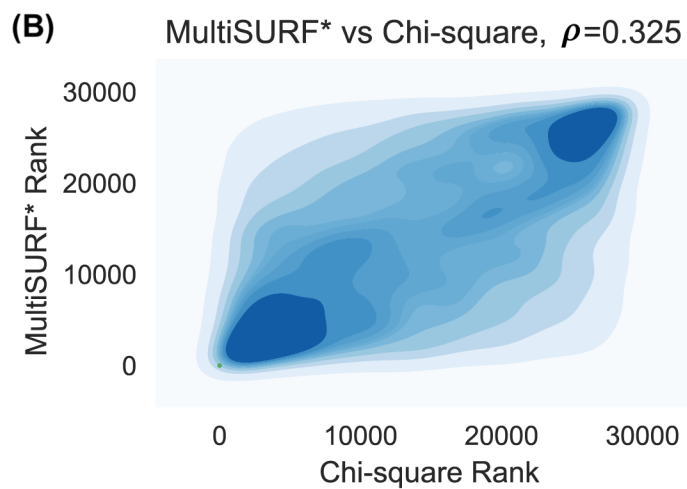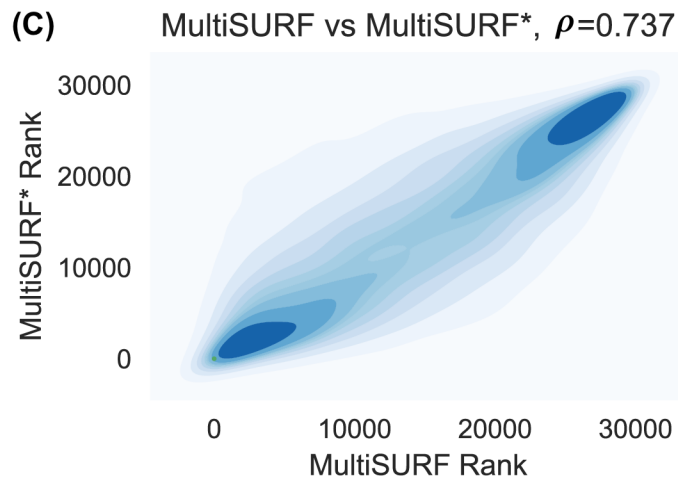

Supplement: Supplementary file 1 — Additional file 1 Density plots depicting the correlation between the gene ranks across the three analyses in Cohort 2. Spearman’s rank-order correlation coefficient (ρ) is given for each comparison. [file 13040_2022_287_MOESM1_ESM.pdf]
